# Ca^2+^ Dependent Formation/Collapse of Cylindrical Ca^2+^-ATPase Crystals in Scallop Sarcoplasmic Reticulum (SR) Vesicles: A Possible Dynamic Role of SR in Regulation of Muscle Contraction

**DOI:** 10.3390/ijms24087080

**Published:** 2023-04-11

**Authors:** Jun Nakamura, Yuusuke Maruyama, Genichi Tajima, Satoshi Hayakawa, Makiko Suwa, Chikara Sato

**Affiliations:** 1 Health and Medical Institute, National Institute of Advanced Industrial Science and Technology, Central 6, 1-1-4 Umezono, Tsukuba 305-8568, Japan; 2 Institute for Excellence in Higher Education, Tohoku University, 41 Kawauchi, Aoba-ku, Sendai 980-8576, Japan; 3 Division of Microbiology, Department of Pathology and Microbiology, Nihon University School of Medicine, 30-1 Oyaguchi-Kamimachi, Itabashi, Tokyo 173-8610, Japan; 4 Biological Science Course, Graduate School of Science and Engineering, Aoyama Gakuin University, 5-10-1 Fuchinobe, Chuou-ku, Sagamihara 252-5258, Japan; 5 Division of Immune Homeostasis, Department of Pathology and Microbiology, Nihon University School of Medicine, 30-1 Oyaguchi-Kamimachi, Itabashi, Tokyo 173-8610, Japan; 6 School of Integrative and Global Majors (SIGMA), University of Tsukuba, 1-1-1 Tennodai, Tsukuba 305-8577, Japan

**Keywords:** scallop, sarcoplasmic reticulum (SR), SR elongation, SR contraction, Ca^2+^-ATPase, calcium, ryanodine receptor, transmission microscopy, cell morphology, cell dynamics

## Abstract

[Ca^2+^]-dependent crystallization of the Ca^2+^-ATPase molecules in sarcoplasmic reticulum (SR) vesicles isolated from scallop striated muscle elongated the vesicles in the absence of ATP, and ATP stabilized the crystals. Here, to determine the [Ca^2+^]-dependence of vesicle elongation in the presence of ATP, SR vesicles in various [Ca^2+^] environments were imaged using negative stain electron microscopy. The images obtained revealed the following phenomena. (i) Crystal-containing elongated vesicles appeared at ≤1.4 µM Ca^2+^ and almost disappeared at ≥18 µM Ca^2+^, where ATPase activity reaches its maximum. (ii) At ≥18 µM Ca^2+^, almost all SR vesicles were in the round form and covered by tightly clustered ATPase crystal patches. (iii) Round vesicles dried on electron microscopy grids occasionally had cracks, probably because surface tension crushed the solid three-dimensional spheres. (iv) [Ca^2+^]-dependent ATPase crystallization was rapid (<1 min) and reversible. These data prompt the hypothesis that SR vesicles autonomously elongate or contract with the help of a calcium-sensitive ATPase network/endoskeleton and that ATPase crystallization may modulate physical properties of the SR architecture, including the ryanodine receptors that control muscle contraction.

## 1. Introduction

Using negative staining and electron microscopy (EM), it was recently observed that the Ca^2+^-ATPase proteins present in SR vesicles isolated from rabbit fast-twitch skeletal muscle form a calcium-sensitive crystalline array in the presence of ATP to elongate the vesicles [1]. However, because the rabbit SR vesicles were highly susceptible to deformation and cohesion/agglomeration in the presence of ATP, it was difficult to obtain sufficient data. To overcome this, a stable SR vesicle preparation with a high calcium transport activity was isolated from scallop [2]. As reported in the previous paper of the scallop SR vesicles [3], the data obtained using this preparation confirm the elongation of vesicles by calcium-sensitive ATPase crystallization previously observed using rabbit SR vesicles. Moreover, an ATPase tetramer was recognized as a structural unit of the crystalline arrays, and ATP was shown to stabilize them. Furthermore, the Ca^2+^-ATPase inhibitor, thapsigargin (TG) [4], was shown to prevent both ATPase crystallization and vesicle elongation. Namely, the experiments confirmed that ATPase molecules gather to form a crystalline array with the help of ATP to elongate the SR vesicles. However, the [Ca^2+^]-dependence of elongated vesicles in the presence of ATP was not studied.

In each scallop-striated muscle cell, SR is a single tubular system in the zone just beneath the cell surface and surrounds only one myofibril at the cell center [5]. When the muscle is relaxed, the SR is a branched tube of uniform diameter [5]. The scallop SR directly associates with the plasma membrane via junctional feet-like structures of ryanodine receptors (RyR; the calcium-release channel of SR) [6,7] (see Figure 1 in Ref. [3]). By contrast, the SR of vertebrate cross-striated skeletal muscle associates with T tubules via the junctional feet-like structures of RyRs [8].

It has been proposed that the Ca^2+^-ATPase molecules of rabbit SR act as a calcium-sensitive membrane-endoskeleton in addition to their primary role as a calcium pump [1]. However, this idea of a membrane-endoskeleton is preliminary; more data is required, especially about calcium sensitivity. On the other hand, an earlier study of scallop ATPase crystallization [9] showed that ATPase crystals collapse when exposed to 0.1 mM Ca^2+^ for 15 min. Considering the widely-accepted concept that the physiological calcium concentration within a cell is ≤10–30 µM, the 0.1 mM Ca^2+^ employed was far above the physiological concentration. A scallop SR preparation with a low calcium transport activity was employed in this earlier study [10]. Here, we use a scallop SR preparation with a high calcium transport activity [2], comparable to the transport activity of rabbit SR preparations, to examine the calcium dependences of ATPase crystallization and elongation of scallop SR vesicles in the presence of ATP. Crystalline arrays of the elongated SR vesicles disappeared as the calcium concentration increased to >18 µM. At this Ca^2+^ concentration, the ATPase molecules were fully active for their calcium transport [2], as are rabbit SR vesicles [1]. Moreover, at the higher calcium concentration, the elongated vesicles transformed into round forms covered by crystal patches, some of which had cracks, probably reflecting the rigidness of the original three-dimensional (3D) spheres. The role of the SR movement is discussed in terms of the regulation of muscle contraction.

## 2. Results

### 2.1. Calcium Dependence of the ATPase Crystallization and Elongation of SR Vesicles in the Presence of ATP

**Figure 1:**
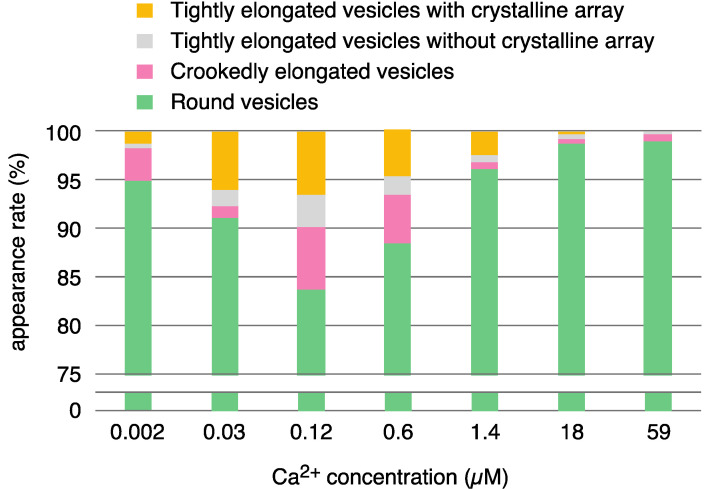
Overview of the appearance rates (%) of various types of SR vesicles relative to the total number of vesicles at ~0.002–59.0 µM Ca^2+^ in the presence of 5 mM ATP. The vesicle classification in Table 1 was simplified to four types: tightly elongated vesicles with one or more crystalline arrays (yellow), tightly elongated vesicles without crystalline arrays (including vesicles with a crystal patch assembly and/or unclear arrays) (gray), crookedly elongated vesicles (pink) and round vesicles (green).

The crystallization of the ATPase molecules was performed according to the procedure, which was described in the previous report on the crystallization [1]. Namely, the SR (0.3 mg of protein/mL) was incubated with 100 mM imidazole buffer (pH 7.0) containing 0.12 M KCl, 5 mM MgCl_2_, ~0.002–59 μM Ca^2+^ with 5 mM ATP at 12 °C for 1 min, unless otherwise indicated. Table 1 and Figure 1 summarize the ATPase particle (~40 Å) dispositions (see Ref. [3] for details) observed in SR vesicles and the vesicular shapes at different calcium concentrations (approx. 0.002–59 µM Ca^2+^) in the presence of 5 mM ATP (see Supplementary Table S1 for full details). The crystalline arrays of ATPase molecules were subclassified into two types: “two-rail” (like a railroad track; see Figure 3c,c’ in Ref. [3]) and monomer (like a lattice of monomers; see Figure 3d,d’ in Ref. [3]). The non-crystalline arrays contained an assembly of various types of small ATPase crystals, including fragmented-ladder and/or “Lego” types, i.e., what we call a crystal patch assembly (mosaic crystallization; see Figure 5a in Ref. [3]). Schematic representations of a “two-rail” crystal array, a monomer crystal array and a crystal patch assembly are shown in Figure 3g,h and Figure 5b, respectively, in Ref. [3].

**Figure 2:**
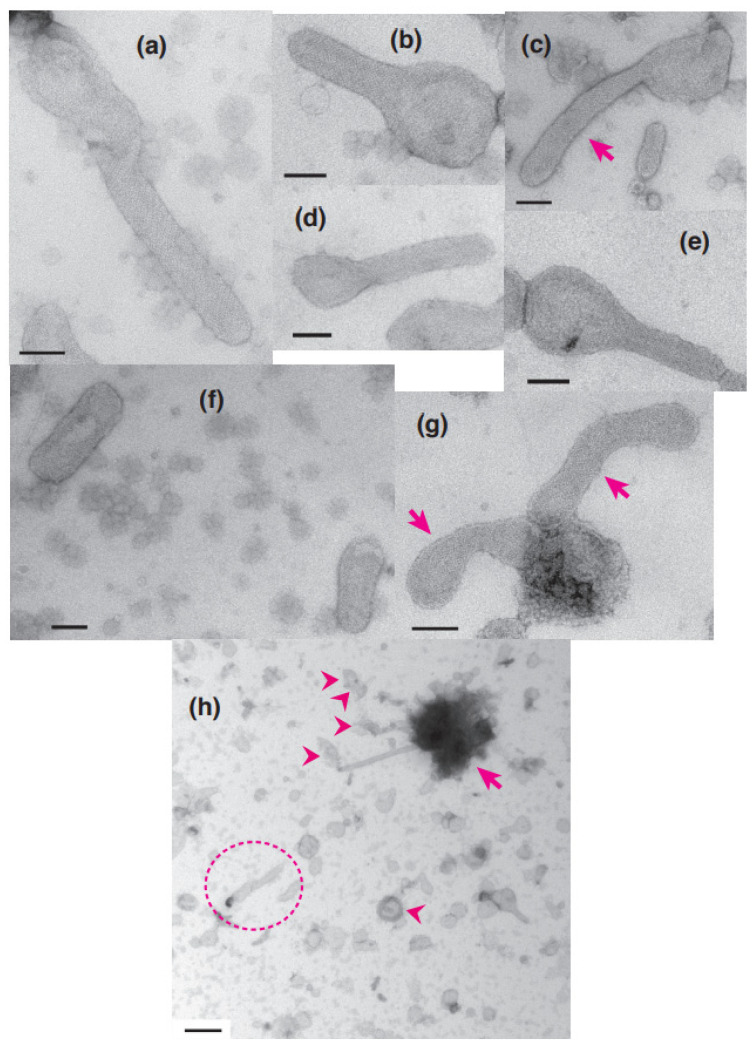
Typical images of elongated SR vesicles with crystalline dispositions and/or crystal patch assemblies of Ca^2+^-ATPase molecules in the presence of 5 mM ATP. (**a**–**c**) Tightly elongated vesicles with a crystalline disposition mainly comprised of two-rail array (marked by arrow). (**d**,**e**) Tightly elongated vesicles with crystalline disposition mainly comprised of monomer array. (**f**) Tightly elongated vesicles with a crystal patch assembly. (**g**) Crookedly elongated vesicles with a crystal patch assembly marked by arrows. (**h**) Overview of vesicles within an electron microscopic view of 5.3 µm by 5.3 µm. Some vesicles contain one or more crystalline arrays; aggregated/conglomerated (arrowheads) and aggregated vesicles (arrow) are indicated. (**a**) is the high magnification image of the vesicle marked with dotted circle in (**h**); the image has been rotated by 90° in the clockwise direction. Scale bars in (**a**–**g**): 100 nm. Scale bar in (**h**): 0.5 µm.

**Figure 3:**
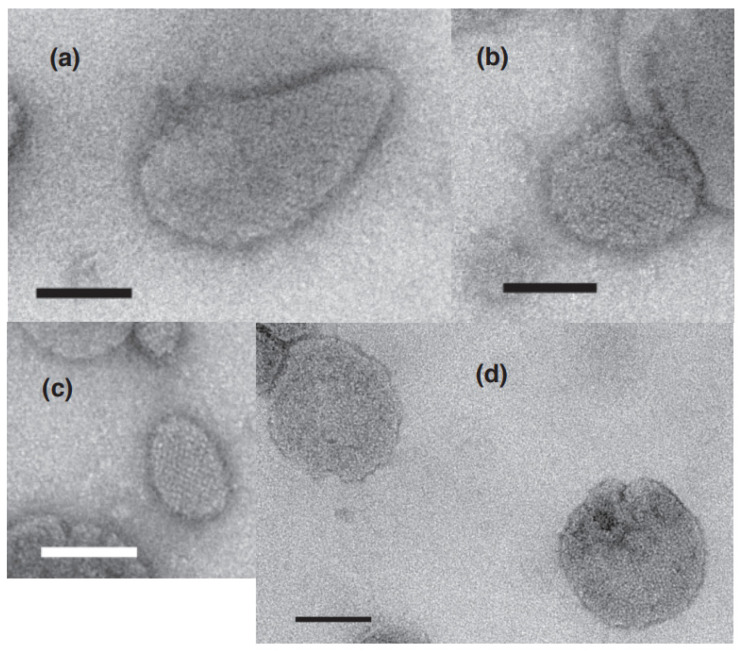
Typical images of the round vesicles with crystalline disposition and crystal patch assembly of Ca^2+^-ATPase molecules in the presence of 5 mM ATP. (**a**,**b**) Round vesicles with crystalline dispositions mainly comprised of two-rail array. (**c**) Round vesicles with crystalline dispositions mainly comprised of monomer array. (**d**) Round vesicles with crystal patch assembly. Scale bars: 100 nm.

Vesicles were classified as elongated or round depending on their shape, the criteria being the ratio of their major axis to their minor axis, ≥2 and <2 for elongated and round vesicles, respectively. The elongated vesicles were further sub-classified into the tightly- and crookedly-elongated types. Figure 2a–c and Figure 3a,b shows typical images of tightly elongated vesicles containing two-rail arrays (at ~0.03 and 0.12 µM Ca^2+^) and round vesicles containing two-rail arrays (at ~0.002 µM Ca^2+^), respectively. Images of elongated vesicles containing two-rail arrays at the very low [Ca^2+^] concentration (0.002 µM) can be found in Ref. [3] (Figure 9a–c). Tightly elongated and round vesicles mainly containing monomer arrays are shown in Figure 2d–e and Figure 3c. The distinct tetragon arrays, observed in the absence of ATP in the previous study of scallop SR vesicles [3], were no longer detected. Crystal patch assemblies were observed in tightly- and crookedly elongated vesicles (Figure 2f,g, respectively) as well as in round vesicles, as shown (Figure 3d). The vesicles with unclear ATPase dispositions in the presence of ATP (data is not shown) and in the absence of ATP (see Figure 5c in Ref. [3]) looked similar.

**Figure 4:**
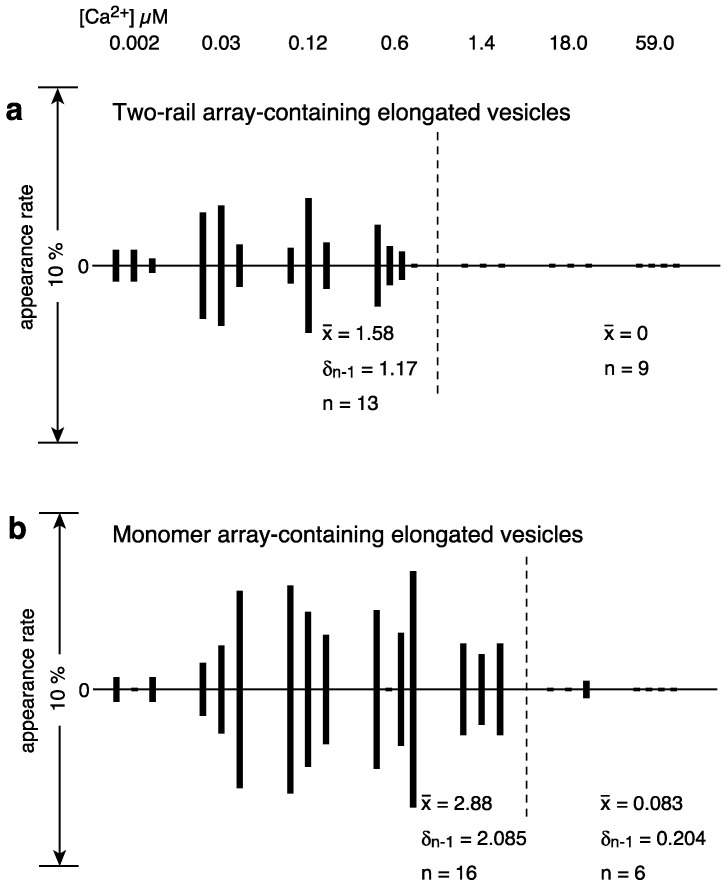
Calcium dependence of appearance rate of tightly elongated vesicles containing two-rail crystal array (**a**) and monomer-crystal array (**b**) in the presence of ATP. Percentages of the number of tightly elongated vesicles with crystalline disposition relative to the total number of vesicles (along the *y*-axis) were plotted versus calcium concentration (along the *x*-axis) (see text for details). (**a**) Elongated vesicles mainly containing two-rail crystalline arrays. (**b**) Elongated vesicles mainly containing monomer crystalline array.

There were 0–17 tightly elongated vesicles with a crystalline array in each view over the calcium concentrations range 0.002–59 µM in the presence of 5 mM ATP, and their average appearance rate (percentage) was at most 6.8%; the crystalline arrays were mainly of the two-rail and/or monomer type, as in Figure 2a–e. Moreover, the percentage of crystal-containing vesicles fluctuated a lot from view to view at any given calcium concentration, the same as in the absence of ATP [3]. Calcium-dependent changes in the Ca^2+^-ATPase disposition and shape of tightly elongated vesicles in the presence of ATP were examined using the method described in the previous study of scallop SR vesicles [3]. Namely, for the tightly elongated vesicles, mainly including the two-rail array, the percentage (see Supplementary Table S1) of the number of vesicles relative to the total number of vesicles (elongated and round) was calculated for each view recorded at each calcium concentration and represented in a vertical amplitude bar graph where the [Ca^2+^] employed was plotted at even intervals from ~0.002 to ~59 μM, along the horizontal axis (see Figure 4a). The amplitude graph of appearance rates (percentages) suggests that the phases when the vesicles were present can be classified into two groups, at ≤0.6 and ≥1.4 μM Ca^2+^, respectively (Figure 4a). At ≤0.6 μM Ca^2+^, the percentage of two-rail-array-containing elongated vesicles relative to the total vesicles (elongated and round) was 1.6% (*SD* = 1.2, n = 13). At ≥1.4 μM Ca^2+^, the percentage was 0% (*SD* = 0, n = 9). On the other hand, elongated vesicles containing monomer arrays appeared up to 1.4 μM Ca^2+^ (Figure 4b); The percentage of monomer-array-containing elongated vesicles relative to the total vesicles was 2.9% (*SD* = 2.1, n = 16) at ≤1.4 μM Ca^2+^. In contrast, the percentage was only 0.08% (*SD* = 0.2, n = 6) at ≥18 μM Ca^2+^ (Figure 4b). These observations suggest that two-rail arrays of the ATPase molecules tend to transform into monomer arrays and then further into other dispositions as [Ca^2+^] increases. The percentage of tightly elongated vesicles containing crystals (mainly two-rail or monomer arrays or their mixture) was 4.2% (*SD* = 2.5, n = 16) at ≤1.4 μM Ca^2+^ and decreased to 0% (*SD* = 0, n = 9) at ≥1.4 μM Ca^2+^.

For rabbit SR vesicles, it was previously reported that the percentage of the number of crystalline-array-containing vesicles relative to the total number of vesicles was about 1.8% at ≤0.09 µM Ca^2+^ in the presence of ATP [1]. The percentage of crystal-containing scallop SR vesicles (~4.0%) reported here is about twice as high. This increase facilitated statistical analysis of the image data. Although both scallop (Figure 2h) and rabbit vesicles (Figure 3a in Ref. [1]) seem to be susceptible to deformation and cohesion in the presence of ATP, scallop vesicles were more stable against deformation and/or denaturation than rabbit vesicles.

The percentages of the number of tightly elongated vesicles containing crystal patch assembly relative to the total number of vesicles were 0.7 (*SD* = 0.8, n = 16) and 0.1% (*SD* = 0.2, n = 6) at ≤1.4 and ≥18 µM Ca^2+^, respectively. Even at ≤1.4 µM Ca^2+^, the percentage was very small, compared with 4.2% (*SD* = 2.5, n = 16) (see Figure 4a,b) for the tightly elongated vesicles containing crystals (mainly two-rail or monomer arrays or their mixture). Crookedly elongated vesicles (Figure 2g) were observed at <1.4 µM Ca^2+^ (Table 1). However, they did not include any crystalline array.

**Figure 5:**
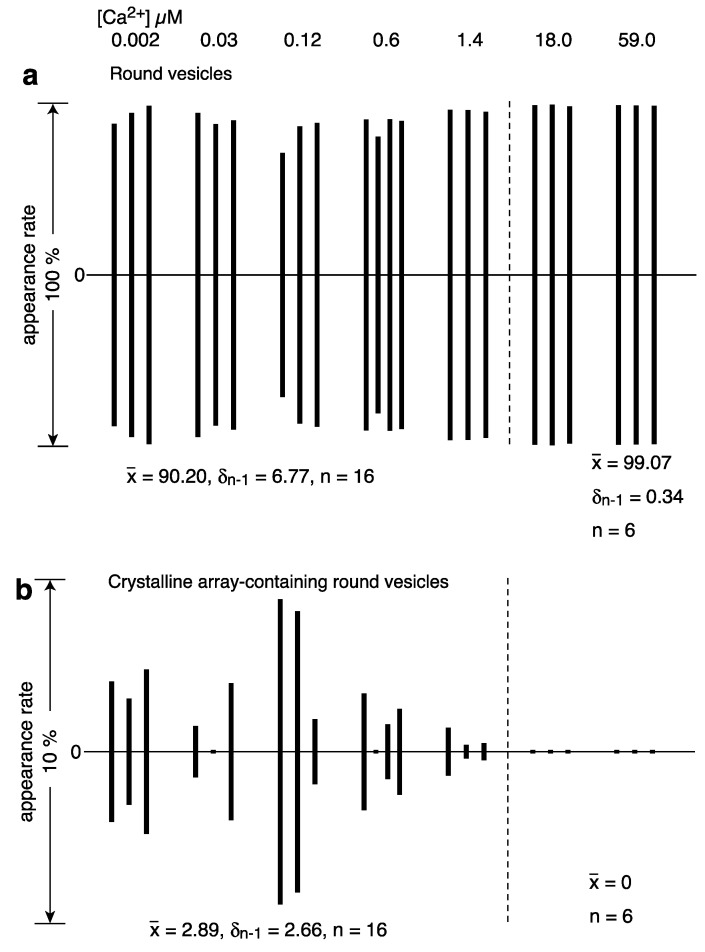
Calcium dependence of appearance rates of round vesicles in the presence of ATP. (**a**) Percentages of the number of round vesicles relative to the total number of vesicles are plotted versus calcium concentration (see text for details). Each bar represents the sum of round vesicles with and without clear crystalline array. (**b**) Round vesicles with two-rail and/or monomer crystalline array.

Round SR vesicles were dominant, independent of the calcium concentration (Table 1). They contained crystalline arrays, crystal patch assembly or unclear arrays. The amplitude graph displaying the percentages of round vesicles relative to the total number of vesicles, however, suggests that the data can be divided into two groups according to the vesicle appearance rate: high (average) 90.2% (*SD* = 6.8, n = 16) at ≤1.4 µM Ca^2+^ and very high (average) 99.7% (*SD* = 0.3, n = 6) at ≥18 µM Ca^2+^ (Figure 5a and Supplementary Table S1).

Among the round vesicles, vesicles containing crystals—mainly two-rail and/or monomer array—appeared at ≤1.4 µM Ca^2+^ and disappeared at ≥18 µM Ca^2+^ (Figure 5b). The percentage of the number of crystal-containing round vesicles to the total number of vesicles was 2.9% (*SD* = 2.7, n = 16) at ≤1.37 µM Ca^2+^ and decreased to 0.00% (*SD* = 0.00, n = 6) at ≥18 µM Ca^2+^ (Figure 5b). This reflects the fact that ATPase crystalline array transformed to crystal patch assemblies at ≥18 µM Ca^2+^ (see Supplementary Table S1 for details). The calcium dependence of the Ca^2+^-ATPase dispositions, including two-rail and/or monomer array, in round vesicles (Figure 5b) was comparable to the dependence found for tightly elongated vesicles (Figure 4a,b) (Supplementary Table S1). However, the size of the various crystalline arrays differed significantly. The array present in round vesicles was generally much smaller than in tightly elongated vesicles. For example, compare the crystals in round vesicles in Figure 3a–c with the crystals in elongated vesicles in Figure 2a–e and in the previous study of scallop SR vesicles, Figure 9a–c [3].

**Figure 6:**
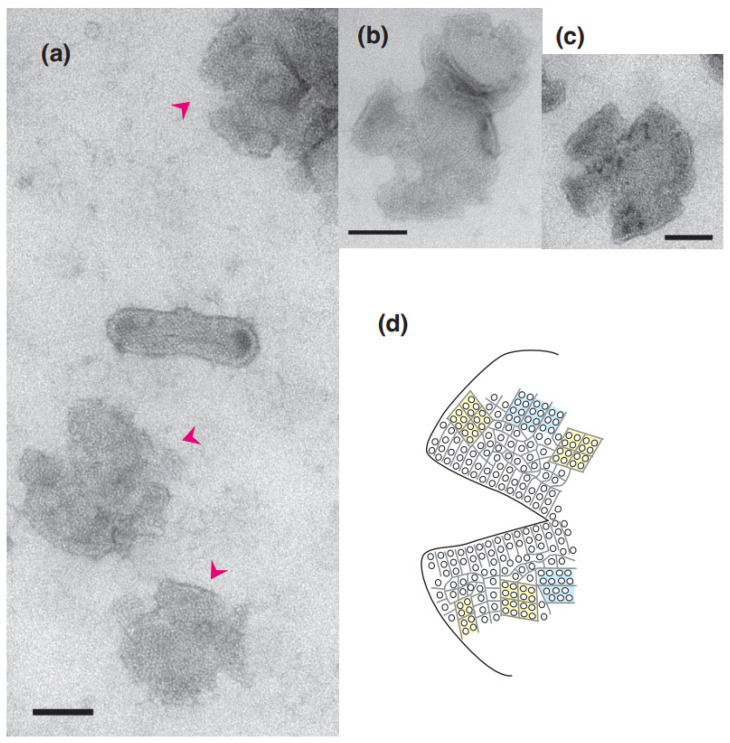
Cracked round vesicles in the presence of ATP. (**a**) Cracked vesicles (marked by arrowheads) at ~0.002 µM Ca^2+^. (**b**,**c**) Cracked vesicles at ~1.4 µM Ca^2+^. (**d**) Illustration of the ATPase disposition inside the crack. Scale bars: 100 nm.

**Figure 7:**
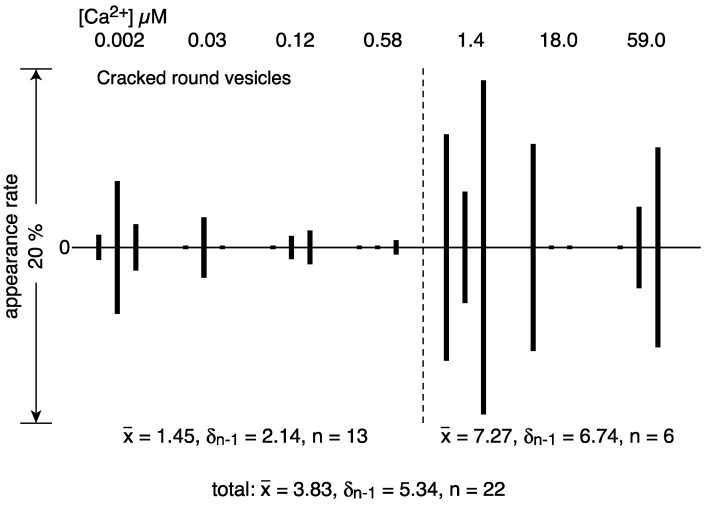
Calcium dependence of the appearance rate of cracked round vesicles in the presence of ATP. Percentages of the number of cracked round vesicles to the total number of vesicles plotted versus calcium concentration (see text for details).

Round vesicles containing ATPase crystal patch assembly sometimes have cracks over the employed Ca^2+^ concentrations in the presence of ATP (Figure 6a–c). The surface of each cracked vesicle was densely covered by patches of tightly aligned ATPase crystalline arrays, especially around the cracks (Figure 6d), although each crystal was small. The amplitude graph depicting the calcium dependence of the appearance rate of cracked vesicles (Figure 7) suggests that the data can be classified into two groups: the average percentage of cracked vesicles relative to the total vesicles was 1.5% (*SD* = 2.1, n = 13) at ≤1.4 and 7.3% (*SD* = 6.7, n = 6) at >1.4 µM Ca^2+^. However, considering the large *SD*, the difference might not be significant; more data are required in order to draw conclusions about a possible calcium dependence of the vesicular crack. Each of the cracked vesicles look like a “cosmos flower” or “Pac-Man” in the TV game. In the absence of ATP, no crack occurred (for example, see Figure 3e in Ref. [3]). It should be noted that such cracks also occasionally occur in the round type of rabbit SR vesicles in the presence of ATP.

### 2.2. [Ca^2+^] Jump-Up and -Down

**Figure 8:**
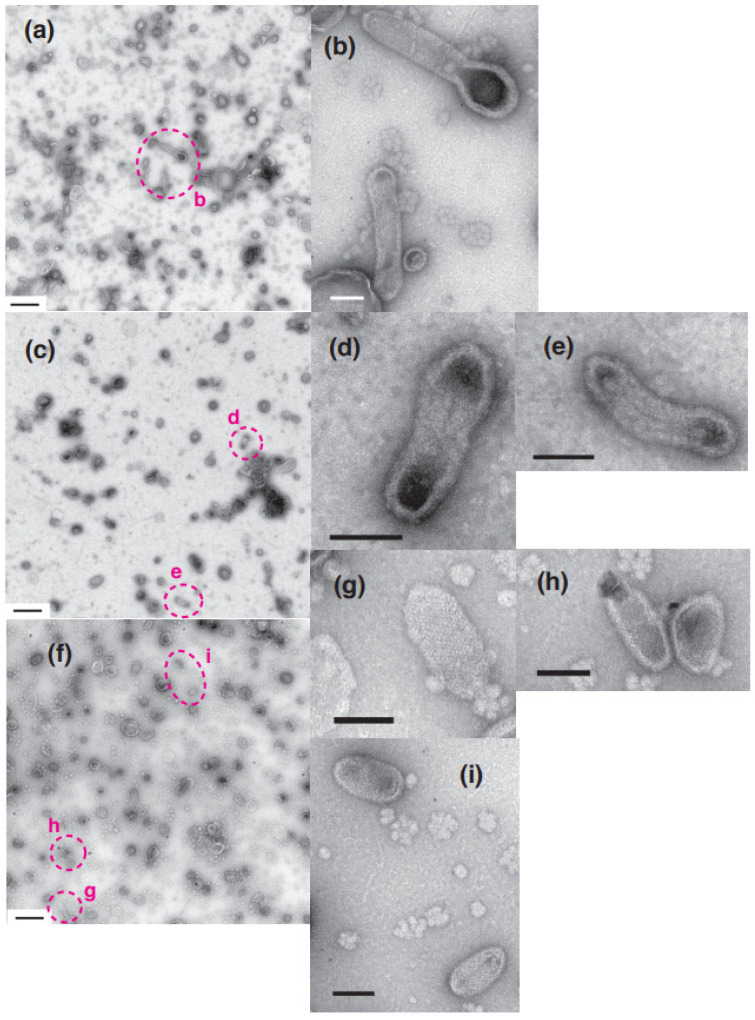
Effect of [Ca^2+^] jump-up on SR vesicles in the presence of ATP. SR preparation (0.3 mg protein/mL) was incubated in buffer solution containing ~0.002 µM Ca^2+^ and 5 mM ATP at 12 °C for 1 min (see “Materials and Methods”). After the incubation, 1/10 volume of water (**a**,**b**) or 28.5 (**c**–**e**) or 20.0 (**f**–**i**) mM CaCl_2_ was added to increase the calcium concentration to ~0.002, ~9.8 (in **c**–**e**) and ~1.1 (in (**f**–**i**)) µM, respectively; the pH of the buffer solutions of (**c**–**e**) and (**f**–**i**) decreased by about 0.2 and 0.1, respectively. The addition of water was carried out as a control for the [Ca^2+^] jump-up experiments. 1 min after the addition of CaCl_2_ or water, a part of the incubation mixture was applied to the electron microscopy grid. (**b**) is the high magnification image of the area (**b**) in (**a**). (**d**,**e**) are the high magnifications of the area (**d**,**e**) in (**c**). (**g**–**i**) are the magnifications of the area (**g**–**i**) in (**f**). Scales bars in (**a**,**c**,**f**): 0.5 µm. Scale bars in (**b**,**d**,**e**,**g**–**i**): 100 nm.

The [Ca^2+^] jump-up experiment from 0.003 to 9.8 µM Ca^2+^ was carried out to examine the response time to a high concentration of Ca^2+^ where the crystallization was scarcely observed (see Table 1 and Supplementary Table S1, Figure 4a,b and Figure 5b). All the five views that were employed showed a rapid collapse of the crystalline array within 1 min after the increase of calcium concentration (Table 2A and Supplementary Table S2, and Figure 8c–e). The decrease in the crystalline array was accompanied by a decrease in the number of elongated vesicles from ~9.8% to ~1.9%. In the control experiment (water addition) of the above-mentioned [Ca^2+^] jump-up, crystalline arrays were still observed. When the upward jump in the [Ca^2+^] was small, i.e., from 0.002 µM to 1.1 µM, crystalline arrays were still observed in tightly elongated vesicles, although the crystallinity might be slightly decreased (Figure 8f–i and Table 2A). The [Ca^2+^] jump-up experiment from 0.003 µM to 1.1 µM was carried out to create doubly sure the observations, shown in Table 1 andSupplementary Table 1, that the crystallization takes place not only at a low calcium concentration of 0.003 µM but also at a higher concentration around 1 µM. The result showed that the crystalline array was maintained after the increase in the calcium concentration to 1.1 µM; it supports the above-mentioned observations of the crystallization at such a higher concentration of calcium. The experiments were repeated to confirm the result. These results also coincide with the observations shown in Table 1 that the round vesicles with a crystal patch assemble become to be predominant at a high calcium concentration of around 10 µM.

**Figure 9:**
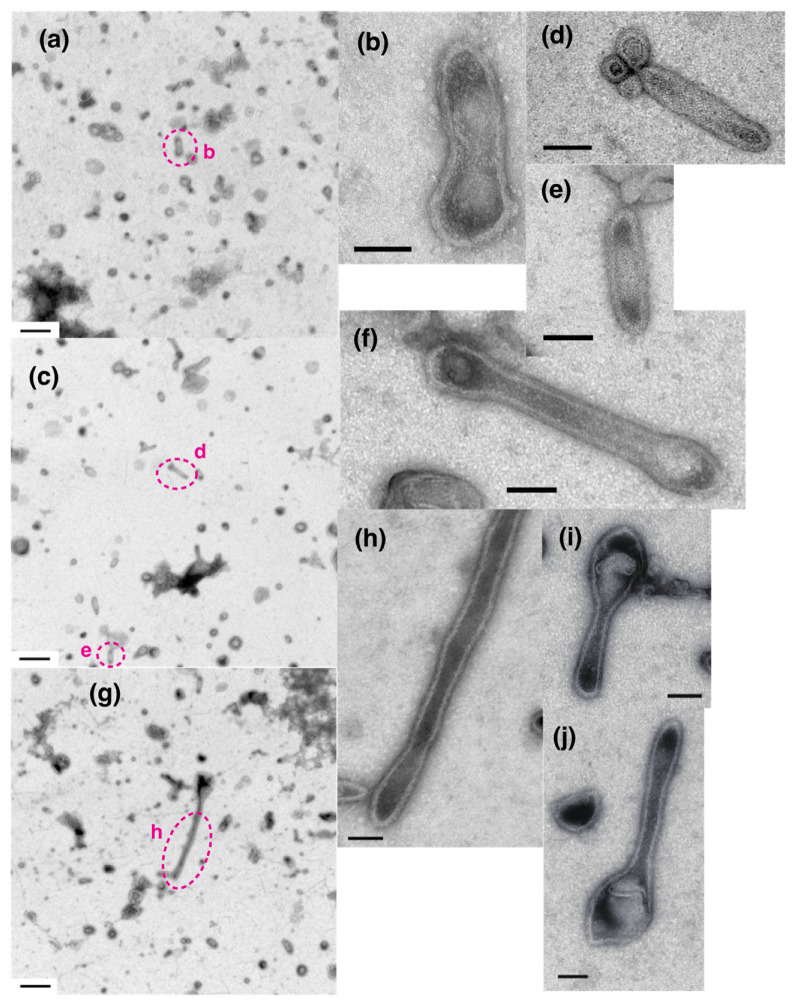
SR vesicles after [Ca^2+^] jump down in the presence of ATP. SR preparation (0.3 mg protein/mL) was incubated in the buffer solution containing ~16.0 µM Ca^2+^ and 5 mM ATP at 12 °C for 1 min (see “Materials and Methods”). After the incubation, 1/10 volume of water (**a**,**b**) or 30.0 mM EGTA (**c**–**h**) was added to the incubation mixture (250 µL). With the addition of the EGTA, the calcium concentration of the reaction mixture jumped down from ~16.0 to ~0.003 µM. The water addition was carried out as a control of the jump-down experiment. 1 min after the addition of water or EGTA, a part of the incubation mixture was applied to the electron microscopy grid. (**b**) is a higher magnification image of the dotted circle (**b**) in (**a**). The images (**c**–**h**) (in (**g**,**h**) is marked with dotted circle) were obtained from the three different areas (see the footnote in Supplementary Table S2 for details). The images (**i**,**j**) were obtained from another jump-down experiment from ~16.0 to ~0.003 µM. Scale bars in (**a**,**c**,**g**): 0.5 µm. Scale bars in (**b**,**d**–**f**,**h**): 100 nm.

After [Ca^2+^] jump-down treatment (Figure 9 and Table 2B), i.e., when the calcium concentration was rapidly decreased from 16.0 to 0.003 µM by adding EGTA solution, most of the elongated vesicles were crooked to a smaller or larger degree (see Figure 9a,c,g). Two sets of jump-down experiments were carried out. The vesicles were, therefore, simply classified into two types: elongated and round. The experiments showed that the percentage of the number of elongated vesicles relative to the total number of vesicles increases within 1 min from ~2.1 to ~14.2% after [Ca^2+^] jump-down (see legend of Table 2 for details). In one of the experiments, a distinct and faint crystal array of ATPase molecules (~40 Å diameter) appeared in three of the elongated vesicles within three views (Figure 9c–f) but not in the control vesicles that were not treated (Figure 9a,b). After the treatment, very long vesicles also appeared, but without a clear crystalline network (Figure 9g,h). In another experiment, however, only one view exhibited two vesicles with only a faint crystal array (Figure 9i,j). Namely, the ratio of the crystalline vesicles observed in the [Ca^2+^] jump-down experiment was small.

In these reversibility experiments, the SR vesicle suspension with a lower or higher concentration of calcium was first agitated after the addition of ATP to start the ATP-supported crystallization. As mentioned above (at p. 7), the agitated scallop SR vesicles exhibited a significant deformation and cohesion, similar to the case of rabbit SR (see Figure 2a in Ref. [1]). After that, the vesicles were further exposed to the second agitation after the increase or decrease in calcium concentration. This repeated agitation seems to cause deadly damage to the vesicles. Especially in the case of calcium jump-down experiments, it was found that (i) the observed numbers of vesicles per view (45 vesicles/5.4 µm by 5.4 µm view, 42 vesicles/3.3 µm by 3.3 µm view (a converted number of the vesicles per 5.4 µm by 5.4 µm ~113), and 42 vesicles/5.4 µm by 5.4 µm view; see Supplementary Table S2 for details)) are significantly small with their ratios of 0.24–0.6 against those of the vesicles that were once agitated in the presence of ATP (179 vesicles/5.4 µm by 5.4 µm view (*SD* = 68, n = 22); see Supplementary Table S1 for details), and (ii) that the background of the obtained electron microscopic (EM) images is deeply stained; the deeply-stained sediment may be composed of the fragmented vesicles. It is probable that the process of the crystal-reversibility test causes deadly damage to the vesicles, resulting in scarcity in the number of observed vesicles.

Considering the above-mentioned damage to the vesicles, the observed crystalline vesicles seem to be survivors, which overcame the difficulty in the crystal-reversibility test. Nevertheless, the small number of images obtained from the results of the [Ca^2+^] jump-up and –down experiments suggest that the ATPase molecules, at least in part, rapidly and reversibly form their crystalline array, depending on calcium concentration.

## 3. Discussion

In their paper addressing the in-situ observation of SR, Castellani et al. [9] report the presence of crystalline arrays of ATPase molecules in intact scallop muscle cells. They propose [11] that, in vivo, such crystal formation might stabilize the scallop ATPase enzyme when the calcium concentration in the sarcoplasm is low, and the muscle is relaxed. On the other hand, from our recent [1,3] and the present studies of the ATPase crystallization in rabbit and scallop SR, we have now accumulated sufficient data to discuss the physiological meaning of the crystallization in detail.

The calcium-dependencies of vesicular shape and their ATPase molecule disposition in vitro, obtained here and in our previous study of scallop SR vesicles [3], can be summarized as follows: In the absence of ATP at ≤1.3 µM Ca^2+^, 1.7% vesicles (*SD* = 1.5, n = 16)) were tightly elongated and contained crystalline ATPase arrays of tetragonal, two-rail and/or monomer units. Crystalline arrays disappeared at ≥19 µM Ca^2+^, and tightly elongated vesicles became less popular (Figure 6 in the previous study [3]). Under these conditions at calcium concentrations between 0.003–69 µM Ca^2+^, most vesicles had round forms (appearance rate of 89.0% on average (*SD* = 7.7, n = 23)) and contained at least one crystal patch assembly (Figure 7 in the previous study [3]).

**Figure 10:**
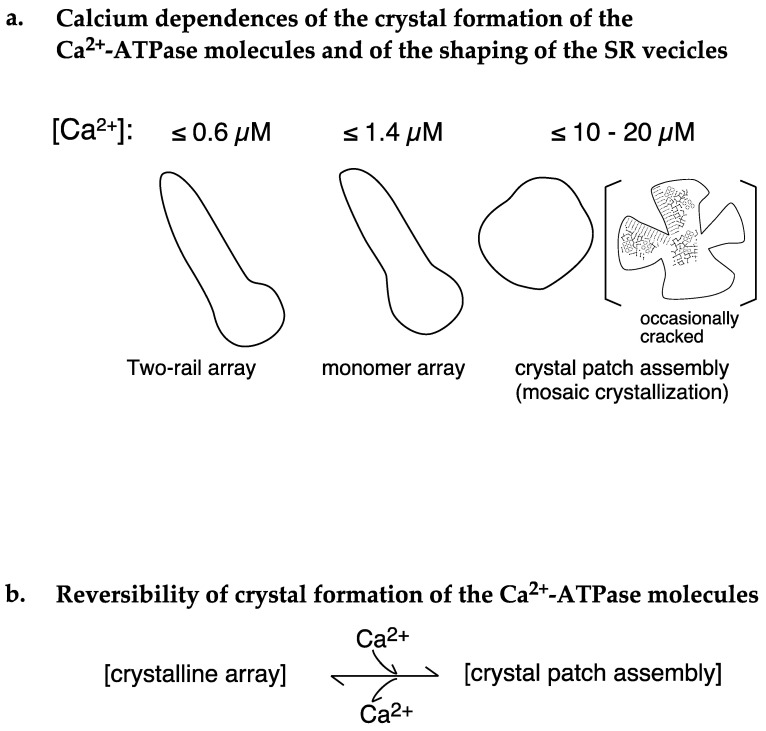
Schematic representation of Ca^2+^-ATPase dispositions on SR vesicles at different [Ca^2+^] in the presence of 5 mM ATP.

**Figure 11:**
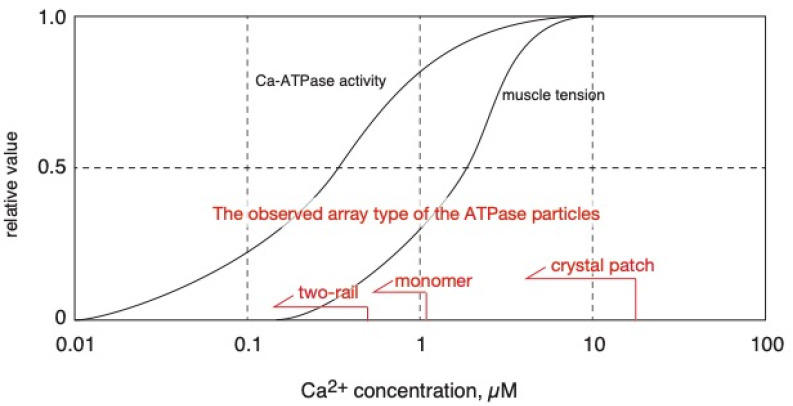
Calcium dependences of Ca^2+^-ATPase activity [2], striated muscle tension development [19] and ATPase disposition (this paper) of scallop in the presence of ATP are schematically represented.

In the presence of ATP at ≤1.4 µM Ca^2+^, (i) 4.2% of the vesicles (*SD* = 2.5, n = 16) had a tightly elongated form and contained crystalline arrays of two-rail and/or monomer units. Further, most of these vesicles disappeared (with the rate of 0.1% (*SD* = 0.2, n = 6)) at ≥18.0 µM Ca^2+^ (Figure 4a,b). (ii) Although round SR vesicles were dominant over the whole calcium concentration examined, their appearance rate can be roughly classified into two groups depending on the calcium concentration: moderately high average appearance rates of 90.2% (*SD* = 6.8, n = 16) at ≤1.4 µM Ca^2+^ and very high average appearance rates of 99.1% (*SD* = 0.3, n = 6) at ≥18.0 µM Ca^2+^ (Figure 5a). (iii) At ≥18.0 µM Ca^2+^, ATPase dispositions on round vesicles are basically a tight-clustering of crystal patches of ATPase (see Figure 3d), and (iv) ATPase crystalline array is rapidly (<1 min) and reversibly formed by the removal of calcium ions (Figure 9a–f) and disappears when 10–16 µM Ca^2+^ is added (Figure 8a–e) (Table 2A,B). The data obtained in the presence of ATP are schematically summarized in Figure 10a,b and Figure 11. Analysis of rabbit SR Ca^2+^-ATPase three-dimensional crystals showed that the ATPase has a modulatory mode of ATP binding [12] in addition to its catalytic mode. ATP binding at the modulatory site of ATPase might have a hydrotropic effect [13] on the ATPase protein to stabilize its structure and reorganize the disposition of the ATPase particles, depending on the calcium concentration.

The crystallization of the scallop ATPase molecules in the presence of ATP was observed earlier [9,10] and is consistent with the observations we made using rabbit SR [1]. Taken together, crystallization seems to be a common role of Ca^2+^-ATPase molecules among vertebrates and invertebrates, although the primary crystal units induced by ATP are slightly different; two-rail and tetramer arrays for scallop and rabbit SR, respectively (Figure 9a–c in Ref. [3] and Figure 3b,c in Ref. [1]). The calcium dependence of the crystallization obtained in the present work was analyzed from static TEM images of SR vesicles on the carbon film of the TEM grid, and their statistics had large *SD*s. However, the data of many independent experiments made using the SRs of scallops and mammals seem to complement each other to depict a common hypothesis for calcium-dependent changes in vesicular shape and their ATPase disposition. For scallop SR vesicles, the data further suggest that a cylindrical ATPase crystal realizes vesicle elongation at lower calcium concentrations (≤1.4 µM) and that this dissociates into tightly clustered patches of small crystals at higher calcium concentrations (10–20 µM), irrespective of presence or absence of ATP, probably inducing the transformation of the vesicles from elongated to round forms.

ATP-treated round vesicles sometimes had cracks (Figure 6a–c) and displayed the following characteristics. (i) Cracks were occasionally observed at the edges of the round SR vesicles for the scallop and the rabbit in the presence of ATP. (ii) Each cracked vesicle was covered by very tightly clustered patches of small ATPase crystals. (iii) Every crack of the vesicles had straight edges that were usually lined by a strict crystal array (Figure 6d and Figure 10a). Since cracks are created when a solid 3D structure is crushed by a large pressure, these results prompt the hypothesis that stable ATPase crystal patches gather close to one another to form a tight assembly, transforming the vesicle into a 3D spherical form. However, when the liquid droplet containing the SR vesicles was dried on the carbon film of a TEM grid, the huge water surface tension pressed and crushed the spherical 3D structure of the SR, as suggested by the crushing of filamentous palladium networks [14] dynamically monitored using our developed Atmospheric SEM (ASEM) [15,16,17,18] as the water droplet containing them dried.

**Figure 12:**
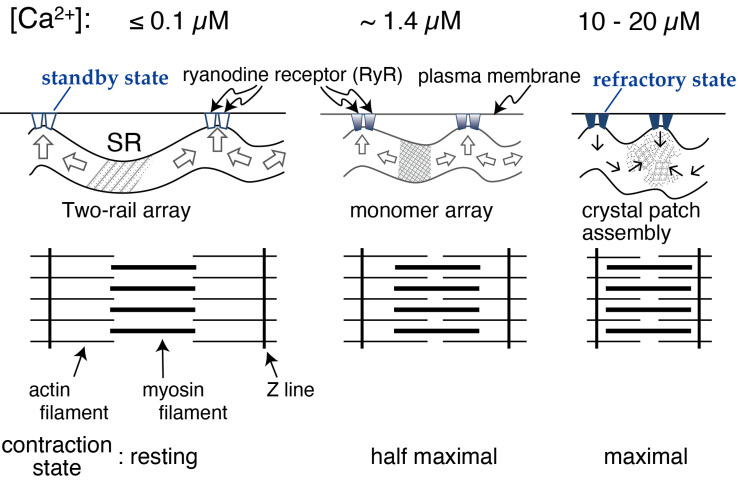
A model linking the transformation of the Ca^2+^-ATPase crystalline array to the contraction of SR in the scallop muscle cell.

The following model and experimental data relating to the calcium transport-coupled movement of the ATPase molecule(s) in the rabbit SR were reported earlier; a model of monomer-dimer transition of the ATPase molecules [20,21,22] and observations of the dynamic, intramolecular movement of the molecule [23,24]. Taking these earlier studies of the rabbit ATPase molecules into account, the calcium-sensitive crystallinity of the scallop ATPase molecules is thought to be indirectly and physically induced by a calcium transport-coupled structural movement within the ATPase molecules. On the other hand, it has been shown that the calcium transport site of the scallop Ca^2+^-ATPase has an apparent calcium affinity (K_0.5_ (calcium concentration for half-maximum ATPase activity) of ~0.3 µM) [2]. Here, we asked whether the appearance frequency of the crystalline patches depends on the ratio of operating ATPase molecules to the total number of ATPase molecules present in the SR vesicles at a calcium affinity (K_0.5_) of ~0.3 µM). The frequency depends on the ratio; the ratios at 0.01, 1.4, and 10–20 µM Ca^2+^ were estimated from the calcium-dependent profile (Figure 11) of the ATPase activity and were almost zero, 0.5, and close to 1.0, respectively. Together with the previous observations, the results prompted us to hypothesize the following molecular mechanism in SR. (i) At [Ca^2+^] ≤ 1.4 μM, the concentration at which the ATPase activity is below its maximum level and muscle contraction is around half-maximum or less, Ca^2+^-ATPase molecules gather to form a cylindrical crystal-network to elongate the SR (see Figure 10 and Figure 11) that is resistant to the water surface tension experienced during sample preparation for EM. The network results in the development of a stretching force in the SR membrane to apply pressure on the nearby mechanosensitive proteins, including ryanodine receptor tetramers (RyRs). These tetramers are known to be Ca^2+^ permeating channels mechanically gated by L-type voltage-sensitive Ca^2+^ channels [25] and to form quasi-crystalline arrays in the triads of skeletal muscle cells and in their isolated forms for vertebrates [26,27,28] and invertebrates [6], and they change their structures [29]. The pressure resulting from Ca^2+^-ATPase crystallization applies pressure on the RyR supercomplexes and puts RyRs in standby mode for electrical depolarization. (ii) At [Ca^2+^] = 10–20 µM, the concentration at which both the ATPase activity and muscle contraction are almost maximum, the elongated SR loses its endoskeleton backbone and contracts (see Figure 10 and Figure 11). (iii) Transformation of the SR into a round form reduces the pressure on the RyRs and puts them in the refractory mode (assuming its existence; see Ref. [30] for the adductor muscle of the human thumb) to work against electrical depolarization of the muscle. In other words, the above discussion suggests that the growth of cylindrical Ca^2+^-ATPase crystals provides a “membrane-endoskeletal motor”, as illustrated in Figure 12.

To supplement the above discussion, it is meaningful to point out that two-rail crystals were observed at lower calcium concentrations, [Ca^2+^] ≤ 0.6 µM (Figure 4a), and monomer crystals at higher calcium concentrations, [Ca^2+^] ≤ 1.4 µM (Figure 4b). Interestingly, 0.6 µM Ca^2+^ is near the calcium concentration (*K*_0.5_~0.3 µM) associated with half-maximum Ca^2+^-ATPase activities [2] and lower than the calcium concentration (*K*_0.5_~2 µM) associated with half-maximal tension development in the scallop muscle [19] (see Figure 11). The transition from two-rail arrays to monomer arrays that takes place between 0.6 and 1.4 µM Ca^2+^ might, therefore, weaken the cylindrical crystal, leading to a decrease in the stretching force of the SR to partially close the calcium permeation pore of the RyR (see Figure 12).

Earlier, it has been shown that each cross-striated adductor scallop muscle cell has a single SR network surrounding only one myofibril [5]. Together with the data reported here, this leads to the following model of the ATPase endoskeleton: The stretching or contractive force developed by cylindrical or mosaic crystallization of ATPase molecules in the single SR network is uniformly transmitted to press or pull all the RyRs, depending on the calcium concentration in the muscle cell (see Figure 12). Because there are tubular SR elements beyond the Z line of myofibrils [31,32,33], a single SR network model is also proposed for a vertebrate cross-striated skeletal muscle cell [31]. This suggests that vertebrate SR might also act as a membrane-endoskeletal motor.

In future studies, cryo-TEM [34,35] might be useful for crystallographic studies of each SR vesicle with the help of caged-ATP, -calcium and -EGTA without drying. The statistical analysis of the SR reported in this and the earlier paper [1] included manual pick-up and classification using human recognition ability. Although both were performed according to strict criteria, a subjective aspect cannot be completely excluded, and, in addition, only a limited amount of data could be analyzed. To create a more precise, completely unbiased data analysis of a very large data set, we are planning to introduce machine learning for image recognition/classification by exploiting artificial intelligence.

## 4. Materials and Methods

Scallop SR was prepared from the cross-striated adductor muscle of the scallop (*Patinopecten yessoensis*) [2], as described in the previous study [3] of scallop SR vesicles.

In the [Ca^2+^] jump-up experiment, SR preparation (0.3 mg protein/mL) was incubated in buffer solution (250 µL) containing ~0.002 µM Ca^2+^ and 5 mM ATP at 12 °C for 1 min, and the incubation mixture was added with 1/10 volume of either water or 28.5 or 20.0 mM CaCl_2_; the calcium additions changed the calcium concentration to ~9.8 or ~1.1 µM, respectively. In the [Ca^2+^] jump-down experiment, SR preparation (0.3 mg protein/mL) was incubated in buffer solution (250 µL) containing ~16.0 µM Ca^2+^, 5 mM ATP at 12 C for 1 min, and the incubation mixture was added with 1/10 volume of either water or 30.0 mM EGTA; the EGTA addition changed the calcium concentration to ~0.003 µM. The water additions in the jump-up and jump-down experiments were always carried out as controls. After 1 min of water addition, CaCl_2_, or EGTA, an aliquot of the incubation mixture was applied to an electron microscopy grid.

Electron microscopic study of the negatively stained SR was performed, also as mentioned in the previous study [3]. The specimens were viewed with a JEM-1230 transmission electron microscope (JEOL, Tokyo, Japan) at 100 kV accelerating voltage.

## 5. Conclusions

The present study has shown that there is a reversible conversion between Ca^2+^-ATPase crystalline array and crystal patch assembly in scallop SR and demonstrated that this is accompanied by the elongation and contraction of the isolated SR vesicles under the physiological conditions of the scallop muscle cell. It has provided some fundamental insights into the active behavior of the scallop and rabbit SR. The data suggest that the tubular scallop SR autonomously elongates and contracts with the help of the calcium-sensitive network of its ATPase molecules, which is called the membrane-endoskeletal motor. The elongation and contraction seem to develop stretching and contractive forces, respectively, in the tubular SR. These forces might regulate the gating of RyRs. The above-mentioned stretching or contractive force, developed in the single solid tubular network of the SR, might be uniformly transmitted to all mechanosensitive RyRs within the cell to engage in the opening, resulting in the unified control of the contraction at the sarcomeres.

## Figures and Tables

**Table 1:** Summarized classification of the SR vesicles at ~0.002–59.0 µM Ca^2+^ in the presence of 5 mM ATP. The table was simplified from Supplementary Table S1 (see text for details). Classification was carried out for the vesicles within 3–4 views of 5.4 µm by 5.4 µm regions, as described in the text. Observed vesicles (major axis > 0.065 µm) were classified as elongated or round types, and the elongated vesicles were sub-classified as vesicles ‘with’ or vesicles ‘without’ a crystalline array of Ca^2+^-ATPase molecules. Numbers in the table indicate the number of each type of vesicle present at each calcium concentration. The number of each type of vesicle relative to the total number of vesicles (elongated and round) at the respective calcium concentrations is given as a percentage in parenthesis. At 0.002–1.4 µM Ca^2+^, the tightly elongated vesicles with and/or without a crystalline array were detected in many views. At 18 and 59 µM Ca^2+^, however, there were only a few elongated vesicles in each view, and sometimes none: At 18 µM Ca^2+^, one view had 3 elongated vesicles while the other two views did not have any. At 59 µM Ca^2+^, two views had an elongated vesicle while the other one did not (see Supplementary Table S1 for details).

			Calcium Concentration (µM)						
			0.002	0.03	0.12	0.6	1.4	18	59
elongated vesicles			32 (5.0%)	45 (9.0%)	68 (16.2%)	48 (11.4%)	30 (3.8%)	8 (1.3%)	5 (1.0%)
	tightly elongated		11 (1.7)	38 (7.6)	41 (9.8)	28 (6.7)	26 (3.3)	3 (0.5)	2 (0.4)
		with crystal-array	8 (1.2)	30 (6.0)	27 (6.4)	20 (4.8)	19 (2.4)	1 (0.2)	0
		without crystal-array	3 (0.5)	8 (1.6)	14 (3.3)	8 (1.9)	7 (0.9)	2 (0.3)	2 (0.4)
	crookedly elongated		21 (3.3)	7 (1.4)	27 (6.4)	20 (4.8)	4 (0.5)	5 (0.8)	3 (0.6)
round vesicles			612 (95.0)	456 (91.0)	352 (83.8)	373 (88.6)	767 (96.2)	618 (98.7)	515 (99.0)
total			644	501	420	421	797	626	520

**Table 2:** Number of elongated and round vesicles in [Ca^2+^] jump-up (A) and -down experiments (B) revealing plasticity of ATPase crystallization and accompanied elongation of the SR vesicles, summarized from Supplementary Table S2. ATPase disposition was simply classified into two patterns, with and without a crystalline array, as described in Table 1. In the [Ca^2+^] jump-down experiment, the SR vesicles were classified into two types: elongated and round vesicles (see text for details). The number of each type of vesicle relative to the total number of vesicles (elongated and round) at the indicated [Ca^2+^] jump-up and -down is given as a percentage in parenthesis. The vesicle populations within electron-microscopic views of 5.4 µm by 5.4 µm were subjected to the vesicle classification, except in one case (see footnote in Supplementary Table S2 for details). The experimental details are described in the text and the legends of Figure 8 and Figure 9.

A: [Ca^2+^] Jump-Up			Control 0.003 → 0.003	0.003 → 1.1 µM	0.003 → 9.8 µM
elongated vesicles			20 (9.8%)	13 (9.0%)	11 (1.9%)
	tightly elongated		20 (9.8)	13 (9.0)	7 (1.2)
		with crystal-array	6 (2.9)	5 (3.4)	0
		without crystal-array	14 (6.9)	8 (5.5)	7 (1.2)
	crookedly elongated		0	0	4 (0.7)
		with crystal-array	0	0	0
		without crystal-array	0	0	4 (0.7)
round vesicles			184 (90.2)	132 (91.0)	550 (98.0)
		with crystal-array	2 (1.0)	2 (1.4)	0
		without crystal-array	182 (89.2)	130 (89.7)	550 (98.0)
total			204 (100)	145 (100)	561 (100)
**B: [Ca^2+^] Jump-Down**			**Control** **16 → 15 µM**	**16 → 0.003 µM**	
elongated vesicles			5 (2.1%)	20 (14.2%)	
		with crystal-array	0	3 (2.1)	
		without crystal-array	5 (2.1)	17 (12.1)	
round vesicles			233 (97.9)	121 (85.8)	
		with crystal-array	0	1 (0.7)	
		without crystal-array	233 (97.9)	120 (85.1)	
total			238 (100)	141 (100)	

## Supplementary Materials

The following supporting information can be downloaded at: https://www.mdpi.com/article/10.3390/ijms24087080/s1.

## Abbreviations

SR	Sarcoplasmic reticulum
EGTA	ethylenebis (oxyethylenenitrilo) tetraacetic acid
TG	Thapsigargin
DMSO	dimethylsulfoxide
TEM	Transmission electron microscopy/microscope
*SD*	Standard deviation
